# Incarcerated Cannulated Cancellous Screw Removal during Total Hip Arthroplasty with a Novel Trick: A Case Report

**DOI:** 10.5704/MOJ.2103.020

**Published:** 2021-03

**Authors:** L Tomar, G Govil, P Dhawan

**Affiliations:** Department of Orthopaedics, Max Super Speciality Hospital Patparganj, Delhi, India

**Keywords:** cannulated screw, hardware removal, hip arthroplasty

## Abstract

Salvage total hip arthroplasty (THA) may be required to manage femoral neck fracture implant failures, avascular necrosis and secondary hip arthritis. Cannulated cancellous screws can become stripped or incarcerated during the initial implantation and pose hardware removal issues.

We present a novel technique for safe screw removal in a 62-year-old female patient with a painful right hip. She had undergone cancellous screw fixation for a fracture of the neck of femur ten years ago. There was avascular necrosis with screw cut out leading to secondary hip arthritis necessitating THA. Intra-operatively cannulated cancellous screw along the inferior femoral neck region was incarcerated. After posterior dislocation of the head, the neck was osteotomised, and the screw threads were exposed for possible extraction. However, the thickened femoral neck region with solid cortical bone prevented the screw disengagement in either direction. The screw along the femoral trochanter region was cut with a Harrington cutter and the remaining screw disengaged with careful removal of bony spicules and controlled anticlockwise rotations, to remove the screw in around fifteen minutes. Arthroplasty could be completed uneventfully thereafter.

We could remove the screw while avoiding an iatrogenic fracture along the calcar region and excessive bone loss along the screw track. The femoral canal remained uncompromised. The anticipation of a difficult implant removal with a thorough understanding of the devices and techniques, is an invaluable asset to the operating surgeon. With a simple tool and novel technique in a difficult situation, we can save on operating time and minimise complications.

## Introduction

Fractures of the femoral neck are difficult to manage. Management techniques by surgical fixation are associated with complications. Avascular necrosis of femoral head, implant failure or screw cut out may lead to secondary arthritis. Recommended treatment option with salvage total hip arthroplasty (THA) gives good functional outcomes.

Removal of hardware may pose a problem in itself^1^. Cannulated cancellous screws commonly used for fixation can become incarcerated or stripped during the process of initial open-reduction and internal fixation or at time of implant removal^2^. An uneventful removal of screw entails short operative time. However, one may have to adapt tricks and tips for better management in difficult situations of screw breakage, stripping or incarceration^3^. Care must be taken to avoid excessive bone loss and iatrogenic fractures during attempts at removal.

We report a case of salvage THA in a 62-year-old female patient with secondary arthritis. An inferiorly placed incarcerated cannulated cancellous screw removal was achieved with a simple novel trick devised per-operatively with a common orthopaedic tool for a successful screw removal and subsequent arthroplasty.

## Case Report

**Fig. 1:**
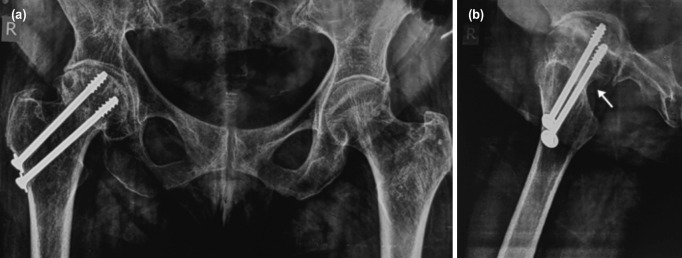
Pre-operative anteroposterior radiograph of both hips (a) with collapse femoral head, implant in situ and screw cut out and lateral radiograph of right hip (b) with screw abutment marked with white arrow at inferior femoral neck region.

A 62-year-old female patient presented with a painful right hip for the last three months. She had no relief with conservative measures. She had a reduction and fixation for a right-sided fracture neck of the femur with two cannulated cancellous screws ten years back. Radiographically there was a loss of femoral head sphericity and superior femoral head cut out with progression to avascular necrosis and secondary hip arthritis (Fig. 1). No signs of infection and no evidence of screw loosening were seen. She consented for a salvage THA.

**Fig. 2:**
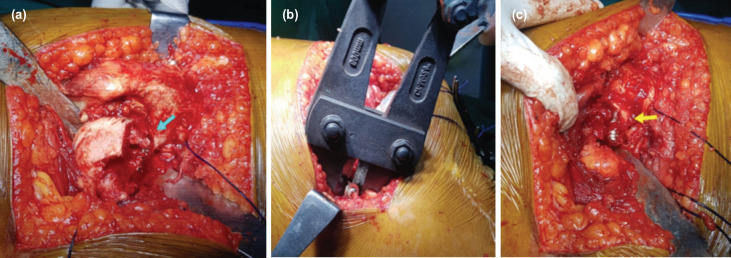
(a) Intra-operatively posteriorly dislocated right hip with exposed screw marked with blue arrow, (b) Harrington cutter for distal screw cut out, (c) screw abutment marked with yellow arrow at inferior femoral neck region.

The patient was operated under a combined spinal and epidural anaesthesia with the affected right hip placed up in lateral position. A posterior approach was used, and the external rotators were tagged. Both cancellous screws were easily identified. The superiorly placed screw with a washer was removed in a routine fashion with a cannulated cancellous screwdriver. The inferior screw was backed out for a distance but was incarcerated, stuck at the region of the distal-most screw thread. Repeated forceful attempts to disengage resulted in the rounding of the screw head. Since a hip arthroplasty was planned, the femoral head was dislocated posteriorly to save on operating time. A neck osteotomy was done at the level based on pre-operative templating. Piecemeal removal of the head was done, and screw threads were exposed (Fig. 2a). However, the screw continued to abut along the neck region with attempts to disengage being unsuccessful.

**Fig. 3:**
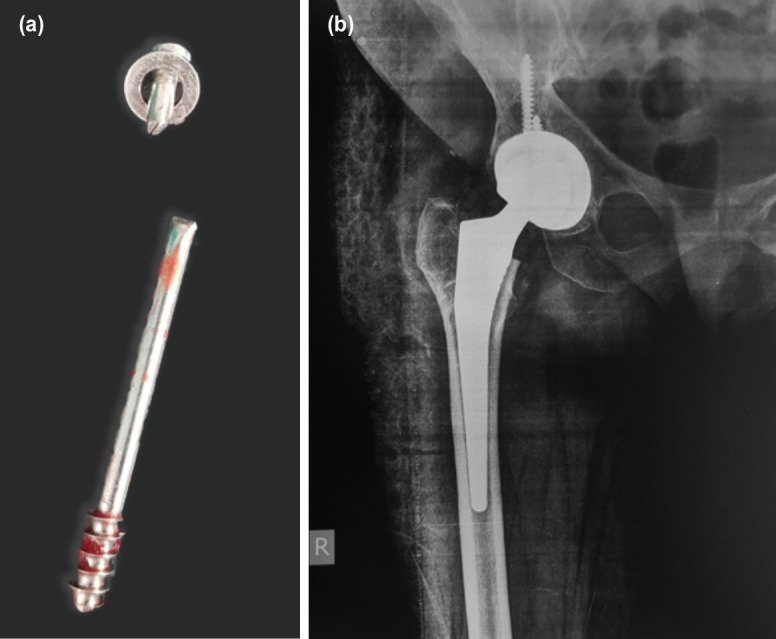
(a) Post-operative clinical photograph of cannulated cancellous stainless-steel screw without metallosis and (b) anteroposterior radiograph right THA.

Extra screw length along the femoral trochanter side was cut with a Harrington cutter (Fig. 2b). The remaining screw was threaded from the neck by carefully disengaging the bony spicules with an anticlockwise rotation and removed as a separate entity (Fig. 2c). The stainless steel screw was removed along with the washer, and there was no evidence of infection or metal deposit in the tissues (Fig. 3a). The time to remove both the screws was approximately fifteen minutes. An uneventful uncemented THA followed with Depuy [Johnson and Johnson, USA] pinnacle acetabular cup with poly-liner and femoral co-rail stem with 32mm ceramic femoral head. Post-operatively the patient was mobilised, painfree with walker support. The post-operative period showed no infection. The immediate post-operative radiograph showed good alignment and cup position (Fig. 3b).

## Discussion

Salvage THA following a proximal femoral fixation failure is a challenging procedure. Hardware removal before THA is associated with high rates of complications^1,3^. Excessive biomechanical and/or shearing stress during repetitive attempts to remove screws during hip arthroplasty may lead to iatrogenic fractures. Systemic osteoporosis and local bone stock reduction due to stress protection are predisposing factors for iatrogenic fractures^1^. The fracture may compound the pre-existing pathology, and there may be a need for revision of plans with an extended surgery time. A revised plan may require a resurfacing or a mini-stem option^1^. With the technique adopted, we could avoid an iatrogenic fracture and excessive bone loss along the screw track. The femoral canal remained uncompromised during the manoeuvre. Obesity may predispose to additional management challenges.

Screw breakage, screw retention, infection, and prolonged operative time are significant problems associated with cannulated cancellous screw removal^2^. Titanium screws have a greater risk of screw head tip stripping and incarceration during removal due to increased osteointegration. Accurate identification of the screw size and manufacturer from conventional radiographs is usually not possible. Implant logs are often unavailable, and screw mismatch with a screwdriver is a major concern^2,3^.

Extensive bone loss secondary to the use of osteotomes, burrs, or intraosseous drilling should be avoided^2,3^. Multiple techniques for cannulated screw removal have been described. Synthes broken screw set’s conical reverse-cutting male-threaded tap is a useful device^3^. Use of a spinal needle to locate a cannulated screw tip and subsequent removal of a buried screw is an alternate technique^4^. A set of sterile Allen keys of different diameters and lengths may be a possible solution^5^. Steinman pin stacked with cement is another option^5^. We used a commonly available orthopaedic tool, the Harrington cutter, and quickly resolved the difficult situation. The aim was to conduct the procedure safely in a short operating time with an eventual good functional result.

A cancellous screw may be incarcerated due to bony ingrowths or abnormal bending. Shear forces at femoral neck probably remodelled the bony trabeculae pattern leading to incarceration. An inferiorly placed cancellous screw should alarm the surgeon for a difficult operative removal.

Implant removal continues to pose challenges for even an experienced surgeon. An understanding of an additional tool and technique which may be used for hardware removal is invaluable for the safe conduction of a difficult salvage THA.
